# Integrity in Physicians

**Published:** 1883-11-20

**Authors:** 


### *INTEGRITY IN PHYSICIANS.*

In all the papers and addresses that are being made upon the subject of Medical Education, and the training of young men for professional life, but little is said upon the importance, on the part of the medical man, of high moral conduct and integrity in his daily walk and life before the world, and of kind relations to professional brethren. If more of this sort of training was inculcated and impressed upon students by preceptors and by the colleges, there would be less talk and less complaint in regard to the violation of the ethics; for the code of ethics, in its true spirit and intent, is but the golden rule, or that physicians, in their conduct toward each other, should simply do as they would be done by.

When, as it sometimes happens in a community where a number of medical men reside, that more than half the number are men of no honesty or integrity of character; no regard for the rights or feelings of medical brethren, and no proper sense of their own reputation or of the dignity and honor of the profession to which they belong, we can expect nothing but jealousies and bitterness to prevail, with injury to the profession and to all who are connected with it in that community. It is policy, not less than correct principle, for physicians to cultivate the most friendly feelings amongst themselves, and so far from opposing or denouncing each other, to pursue invariably the opposite course; talk for each other, defend each other, and help each other in every possible and honorable way. If they will thus act, they will have more practice, more fees for consultations, will make more money, and by frequently mingling and talking with each other will the better enjoy life, and by the interchange of ideas and views make greater progress and accomplish more good in the practice of the profession.

